# Cell Therapy in Organ Transplantation: Our Experience on the Clinical Translation of Regulatory T Cells

**DOI:** 10.3389/fimmu.2018.00354

**Published:** 2018-02-26

**Authors:** Niloufar Safinia, Nathali Grageda, Cristiano Scottà, Sarah Thirkell, Laura J. Fry, Trishan Vaikunthanathan, Robert I. Lechler, Giovanna Lombardi

**Affiliations:** ^1^Department of Immunoregulation and Immune Intervention, Faculty of Life Sciences & Medicine, King’s College London, London, United Kingdom; ^2^Faculty of Medicine, Division of Digestive Disease, Imperial College London, London, United Kingdom; ^3^Clinical Research Facility GMP Unit, NIHR Biomedical Research Centre at Guy’s and St Thomas’ NHS Foundation Trust and King’s College London, London, United Kingdom; ^4^The Blizard Institute of Cell and Molecular Science, Queen Mary University of London, London, United Kingdom

**Keywords:** transplantation, regulatory T cells, clinical trials, good manufacturing practice, cell therapy, technical transfer

## Abstract

Solid organ transplantation is the treatment of choice for patients with end-stage organ dysfunction. Despite improvements in short-term outcome, long-term outcome is suboptimal due to the increased morbidity and mortality associated with the toxicity of immunosuppressive regimens and chronic rejection (1–5). As such, the attention of the transplant community has focused on the development of novel therapeutic strategies to achieve allograft tolerance, a state whereby the immune system of the recipient can be re-educated to accept the allograft, averting the need for long-term immunosuppression. Indeed, reports of “operational” tolerance, whereby the recipient is off all immunosuppressive drugs and maintaining good graft function, is well documented in the literature for both liver and kidney transplantations (6–8). However, this phenomenon is rare and in the setting of liver transplantation has been shown to occur late after transplantation, with the majority of patients maintained on life-long immunosupression to prevent allograft rejection (9). As such, significant research has focused on immune regulation in the context of organ transplantation with regulatory T cells (Tregs) identified as cells holding considerable promise in this endeavor. This review will provide a brief introduction to human Tregs, their phenotypic and functional characterization and focuses on our experience to date at the clinical translation of Treg immunotherapy in the setting of solid organ transplantation.

## Tregs: Phenotype and Heterogeneity

Tregs are characterized by the expression of CD4 and CD25 molecules and more specifically the transcription factor FOXP3, a master control gene underpinning Treg development and function. More recently, the inverse expression of the α-chain of IL-7R, CD127, combined with the expression of CD4 and CD25 has been shown to demarcate a pure population of Tregs demonstrating stability and optimal function (10, 11). Tregs are far from homogenous and over the years have been characterized into several subsets, most of which have been associated with various facets of Treg function (12).

Treg subsets can firstly be stratified according to their origin: thymus-derived and peripherally derived Tregs (tTregs and pTregs, respectively). While there have been phenotypic markers which have suggested differentiation between the two subsets, such as Helios and Neuropilin-1, to date these are still contentious (13–16). The only way to reliably differentiate tTregs and pTregs has been to interrogate the Treg-specific demethylated region (TSDR). Here, tTregs display a fully demethylated TSDR, whereas pTregs are only partially demethylated (17–20). Furthermore, Tregs have also been delineated on account of their antigen naivety in relation to their differential expression of CD45RA and FOXP3. The seminal work of Miyara et al., described the heterogeneity of the Treg population in three phenotypically and functionally distinct subpopulations: Population I (P1) naive or resting Tregs (CD45RA^+^FOXP3^Lo^); population II (P2) effector Tregs (CD45RA^−^FOXP3^Hi^), both of which are suppressive *in vitro*; and population III (P3) the “non-suppressive,” cytokine secreting non-Tregs (CD45RA^−^FOXP3^Lo^) (21). However, we and others have previously shown that population III is indeed suppressive, and within this population, identified a subpopulation of Tregs that express the C-type lectin CD161 and produce the proinflammatory cytokine, IL-17 (22–24).

In support of the heterogeneity of the Treg compartment, using a new technology, the single-cell mass cytometry (cytometry by time-of-flight) we have conducted an in-depth characterization of Tregs, further demonstrating the true extent of their heterogeneity, with 22 different clusters identified (25). In the clinical setting and through utilizing the same principles and technology, Kordasti et al. identified the Treg subset that predicted response to immunosuppressive therapy in patients with aplastic anemia (26). Additionally, we have recently extended the analysis of T helper-like subpopulations of Tregs, demonstrating that Th2-like Tregs are enriched in the tumor sites (27). There is no doubt that the future will see the discovery of many more markers heralding Treg purity. For an in-depth review into Treg phenotype subsets the reader is directed to the following reviews (12, 28, 29).

## Treg Mechanism of Action

Tregs are defined by their immunoregulatory suppressive qualities. However, no one mechanism defines Tregs. Instead, it is believed that several mechanisms behave in concert, which promote immune regulation. All T lymphocytes rely on IL-2 for their survival and proliferation. By their expression of the interleukin 2 receptor, CD25, Tregs deplete stores of IL-2, curbing the survival of surrounding T lymphocytes (28). In a more active mechanism of suppression, the Treg surface molecule, cytotoxic T lymphocyte antigen 4 (CTLA-4), is known to bind the costimulatory molecules CD80/86 with a higher affinity than its proinflammatory competitor CD28, expressed on conventional T effector cells, thus preventing T effector activation. This negative costimulatory molecule has also been proposed to upregulate indoleamine 2, 3-dioxygenase expression on dendritic cells, responsible for the catabolism of tryptophan, which in turn suppresses immune responses through the generation of the immunosuppressive molecules, in particular kynurenine (30). Further in-depth investigation into the mechanism of action of CTLA-4 has revealed that CD80/86 ligands on antigen-presenting cells (APCs) are captured by a process of trans-endocytosis, in turn impairing further T cell activation (31–33). Deficiencies in CTLA-4 have been associated with lymphoproliferative disorders and the development of severe T-cell-mediated autoimmune diseases, which is why CTLA-4 is recognized as a key molecular target governing Treg-mediated suppression (33, 34).

The ectoenzyme, CD39, is abundantly expressed on Tregs, its expression allows Tregs to hydrolyze the proinflammatory danger signal, adenosine triphosphate, to the anti-inflammatory mediator, adenosine, which following interaction with the adenosine A2A receptor, has been reported to have immunosuppressive and anti-proliferative effects (35). CD39^+^ Tregs have also been found to suppress the release of IL-17, alongside IFN-γ and IL-2, from Th17 cells, while CD39^−^ T cells had an increased propensity to produce IL-17 (36). Furthermore, the reconstitution of positively selected CD39-null mouse models of colitis with soluble apyrase, a mediator with enzymatic activity identical to CD39, reversed their increased susceptibility to develop auto-immune diseases and prevented a Th-1 skewed immune response. Further studies by Gibson et al., have highlighted the importance of CD39 expression for Treg mechanism of action in a T cell transfer model of colitis (37). Additionally, we have established a further mechanism by which Tregs function through the release of exosomes, expressing ectoenzyme CD73, which regulate target cells through the purinergic generation of adenosine (38).

Reports have also suggested a cytotoxic role of Tregs in depleting T effector numbers through a perforin-dependent and granzyme-dependent manner (39). Similarly, there have been reports of Tregs expressing Galectin-9, which following binding with the T-cell immunoglobulin and mucin-domain containing-3 receptor CD44 on effector T cells, has been shown to induce apoptosis (40). More recently, this mechanism of suppression has been proposed to be limited to pTregs (41). It has also been postulated that Tregs have the capacity for cytokine-mediated suppression involving the regulatory cytokines: IL-10 (42), TGF-β (43, 44), and more recently IL-35 (45).

## Treg Isolation and Expansion

Clinical trials of Treg therapy in transplantation are focused on tipping the balance of immune homeostasis in favor of regulation. However, in order for this to occur the *in vivo* Treg pool needs to be expanded significantly in order to drive immune tolerance (46). As such, there has been huge interest in either the *in vivo* expansion of these cells or their adoptive transfer. Here, we focus on the prerequisites that need to be fulfilled in order to permit the adoptive transfer of these cells.

Firstly, Tregs need to be isolated from the peripheral blood and this is by no means an easy feat. As mentioned earlier, Tregs are highly heterogenous and as such, the debate of which population of Tregs would serve as optimal cell product in adoptive transfers is a highly discussed topic. Isolation to date has largely been governed by magnetic bead isolation, a process whereby depletion of CD8^+^ T cells and positive selection of CD25^+^ T cells using magnetic beads allows isolation of Tregs (47, 48). This process results in the separation of a consistent number of cells from whole blood using an approach that has been utilized by us in the two clinical trials aimed at testing the safety of expanded autologous Tregs in patients receiving either kidney (ONE Study, NCT02129881) or liver (ThRIL, NCT02166177) transplants. However, this process does not allow for enrichment based on multiple parameters and isolating Tregs based upon CD25^+^ expression alone results in contaminating T effector cells which express low levels of CD25 molecules. Methods to overcome this have been explored in Treg expansion. One such strategy is the *ex vivo* expansion of these cells in the presence of the mTOR-inhibitor, rapamycin (RAPA) (49).

We and others have shown that RAPA is an ideal treatment strategy for preferential expansion of Tregs (50–52). RAPA confers a proliferative advantage to Tregs by affecting the Akt/mTor pathway. Indeed, the molecular signal controlled by this pathway is not essential for Tregs, but crucial for the activation and proliferation of conventional T cells (51, 53). Using this approach, we have shown that large-scale expansion of functionally potent Tregs is possible when starting from a population of cells with low purity (48). In our studies, another potential candidate drug for the expansion of Tregs is the vitamin A metabolite, all-trans retinoic acid (ATRA). Some studies have shown that ATRA can be a good treatment for the induction of adaptive Tregs (54) or very pure Tregs (55). Our data showed that ATRA favors the *ex vivo* expansion of a population of highly suppressive Tregs from magnetic bead purified Tregs, although these cells produce a significant amount of IL-17 and IFN-γ following stimulation. However, further characterization of Tregs cultured with ATRA has shown the expansion of FOXP3^+^CD161^+^ Tregs (51), which is encouraging based on our previous work reporting that CD161 identifies a specific sub-population of IL-17-producing FOXP3^+^ Tregs with a strong capacity to suppress conventional T cell proliferation (22). In contrast, Tregs expanded in the presence of RAPA show decreased expression of CD161, as well as reduced production of pro-inflammatory cytokines. The combined treatment of Tregs with RAPA and ATRA demonstrated that the suppressive function and stability of Tregs is maintained (decreased CD161 expression and lack of IL-17 production).

This analysis was extended to the subtypes of Tregs and while P2 Tregs did not expand *in vitro*, P1 Tregs expanded well in the presence of RAPA, ATRA or the combination of the two drugs. In contrast, RAPA (alone or in combination with ATRA) strongly reduced P3 Treg proliferation, while the treatment with ATRA alone showed a negligible inhibitory effect on the expansion of this subset (51). In support of this observation, we have unpublished data showing that when P1 and P3 were cultured together at 1:1 ratio, the presence of RAPA, but not ATRA, gave a much stronger proliferative advantage to P1 on P3 Tregs. At the end of culture, P1 could overgrow P3 Tregs. Additional experiments showed that RAPA treatment could inhibit the production of proinflammatory cytokines in the P3 subset and negatively affect the expansion of FOXP3^+^CD161^+^ Tregs (51). Finally, we have also demonstrated that these treatments can influence the migratory ability of expanded Tregs. Our findings showed that the treatment of Tregs with RAPA led to the expression of skin-homing receptors cutaneous lymphocyte-associated antigen and CCR4 (51), as well as CXCR3, a chemokine receptor which enables homing to the liver (48). Instead, Treg cultures in the presence of ATRA resulted in a high percentage of cells coexpressing gut-homing receptors such as CCR9 and α4β7 (51).

## Technical Transfer of the Treg Manufacturing Process into the Good Manufacturing Process (GMP) Unit

One of the most difficult and time-consuming aspects of translating the extensive research carried out in the laboratory to the clinical setting is the transfer of research protocols into a GMP unit. This process, referred to as technical transfer, requires careful management to ensure the resulting product maintains a consistently high level of quality, while achieving the aim of the researcher. In this pursuit, a manufacturing process was developed with the aim of creating a Treg Investigational Medicinal Product (IMP) that could be used in the ONE Study (NCT02129881) or ThRIL (NCT02166177) clinical trials.

**Figure 1:**
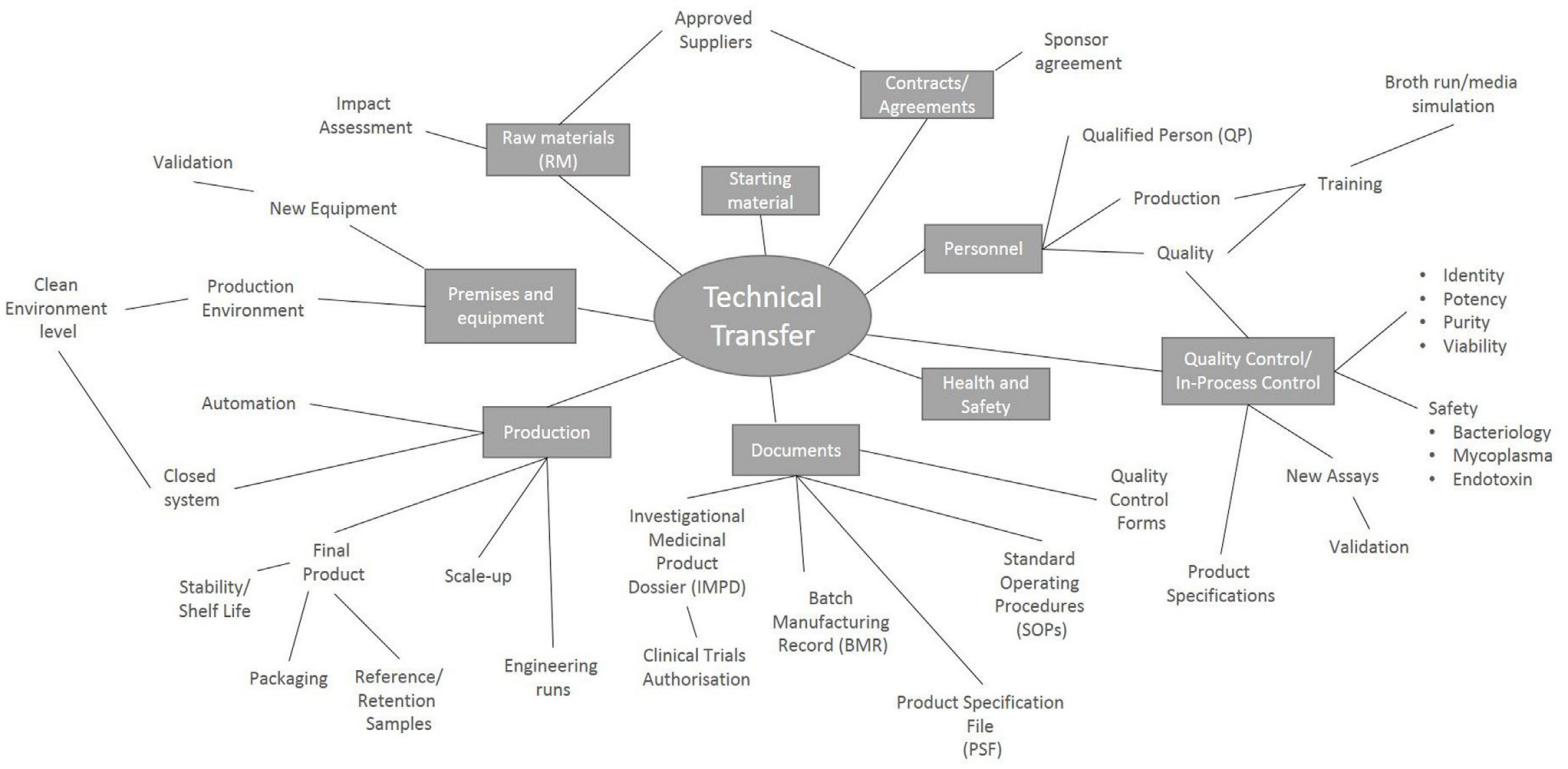
Key points to consider during a technical transfer process. Schematic representation of the processes involved during the transfer of a manufacturing process from the research laboratory into a Good Manufacturing Process unit.

There are many factors to consider during the technical transfer (Figure 1), and important milestones include: the sharing of technical information from the research department to the GMP facility, process development, preparation of the product specifications, process validation and finally authorization of the process by the Medicines and Healthcare Products Regulatory Agency (MHRA) through the submission of a Clinical Trials Authorization (CTA). The process initially starts with the sharing of a detailed description of all process raw materials, manufacturing methods, equipment used, specifications, and test methods from the transferring research department to the GMP unit. It is important to ensure that all raw materials, reagents and consumables that were used in the research laboratory are GMP-compatible. During the technical transfer stage of these Treg trials, different GMP-compatible reagents and culture conditions were compared, ensuring that the final protocol resulted in phenotypically stable Tregs that could be isolated and expanded consistently while maintaining their suppressive function (Table 1).

**Table 1:** Comparison of research- and GMP-grade reagents.

Reagent	Research lab		GMP unit	
CD8 MicroBeads	CD8 MicroBeads	Miltenyi Biotec	CliniMACS CD8 Reagent	Miltenyi Biotec
CD25 MicroBeads	CD25 MicroBeads	Miltenyi Biotec	CliniMACS CD25 Reagent	Miltenyi Biotec
αCD3/CD28 Beads	Dynabeads™	Invitrogen	GMP ExpAct Treg Beads	Miltenyi Biotec
Interleukin-2	Proleukin	Novartis Pharmaceuticals	Proleukin	Novartis Pharmaceuticals
Rapamycin	Rapamycin	LC Laboratories	Rapamune	Pfizer
Cell culture medium	X-Vivo 15 with Phenol Red	Lonza	TexMACS	Miltenyi Biotec
Human AB serum	Research grade	Biowest	Premium grade	Seralab
Expansion device	Culture plates and flasks	VWR	Culture bags	Miltenyi Biotec

The transfer of the Treg process into the GMP unit also provided the opportunity to optimize the manufacturing method and introduce automated processes to increase the levels of consistency between batches. The original research process included a peripheral blood mononuclear cells (PBMCs) isolation using density gradient centrifugation with Ficoll^®^ Paque Plus (GE Healthcare, Switzerland) and 50 ml centrifuge tubes. In order to close out and automate this process, the SEPAX system (Biosafe, Switzerland) was adopted. The SEPAX device is an automated cell separation system which processes within closed single-use disposable tubing sets. The maximum blood input volume to run the NeatCell Programme (SEPAX density gradient cell separation using Ficoll^®^ Paque Plus) is 120 ml, therefore the SEPAX volume reduction programme (SmartRedux) was introduced to ensure the full initial blood sample could be processed. The data generated from these initial runs identified that the volume reduction step alone was sufficient, as the erythrocytes did not interfere with the CliniMACS Plus (Miltenyi Biotec, Germany) magnetic cell selections.

The specifications of the final product are set by the research team and from this information, a product specification file (PSF) can be written. The PSF contains, or refers to files containing, all the information required to draft the standard operating procedures, batch manufacturing records, and the quality control assay forms. These documents ensure standardization and traceability of the process for every batch manufactured.

With these documents in place, scale-up runs can be performed allowing the manufacture process to be tested within the GMP unit and ensuring enough Tregs could be generated to produce the dose required for the patients, perform all QC assays and to create the required reference and retention samples. During the scale-up runs, the opportunity to further close out the system and minimize the risk of contamination was taken. For example, Tregs were cultured using plates and T-flasks in the research laboratory. During the scale-up process, this method was adapted to utilize closed-system culture bags. However, it was found that transferring the process into bags reduced the expansion rate of the Tregs. In spite of this, the required numbers of Tregs were still achieved and so this closed system modification was incorporated into the final manufacturing process.

With the manufacturing methods finalized during the scale-up runs and following the validation of the QC assays, the entire process was validated by performing six engineering runs. These engineering runs provided documented evidence that the GMP unit could routinely and consistently produce the Treg product that met the required specifications.

The data, from both the research experiments and GMP unit, was compiled to create the IMP Dossier (IMDP). This document contains information on the quality, manufacture and control of the IMP. This is one of the essential documents, along with a EudraCT number and approval from an ethics committee, that form the CTA submission to the MHRA.

## Recruitment and Manufacture of Treg Batches in the One Study and ThRIL Trial

The ONE Study (NCT02129881) initiated in 2014, a dose-escalation phase I/II trial carried out under a large EU consortium. The aim of The ONE Study was to evaluate different regulatory cells in kidney transplant recipients allowing the direct comparison of the safety, practicality and therapeutic effect of each cell type. In the UK, in collaboration with The University of Oxford, our group manufactured sixteen batches of polyclonal Tregs, of which 12 were certified for administration. A dose escalation design was implemented with four escalating doses of 1 × 10^6^, 3 × 10^6^, 6 × 10^6^, and 10 × 10^6^/kg, with the last patient being treated with the maximum dose in January 2016. Four of the batches could not be certified and dosed, three of which were due to insufficient cell numbers to formulate the dose and one batch failing the release criteria due to bacterial contamination. High levels of variability were observed in the expansion capability of the Tregs obtained from different patients. Due to this, a substantial amendment was made to the IMPD allowing flexibility in setting a date for the harvesting of Treg culture. This amendment reduced the risk of failing batches during the manufacture of an expensive and time-consuming product.

The last patient in the ThRIL trial (NCT02166177) was dosed in July 2017. This was a Phase I/IIa clinical trial of Treg immunotherapy in the setting of liver transplantation initiated at King’s College London. Here, the safety, tolerability and efficacy of polyclonally expanded Tregs in combination with depletion of alloreactive T cells (ATG) and short-term immunosuppression was assessed. For this trial, patients awaiting a liver transplant were recruited with the aim of administering the cell therapy 2 months after transplantation. Out of an initial 23 patients enrolled, 7 of these patients received a transplant and the manufacture of the Treg IMP was initiated. Of these seven batches, three were completed and certified by a qualified person for administration. Two patients were removed during manufacturing, one due to proteinuria and the other due to death unrelated to the transplant. The other two batches did not meet the specifications (cell number and purity). The complications in patient retention lead to a substantial amendment to the MHRA to allow the recruitment of patients 6-month posttransplantation rather than while they were on the waiting list. This delay in recruitment was made as the transplanted patients were deemed to be more stable, and hence more amenable for cell therapy application. After this amendment was approved, a further six batches of the Treg IMP were manufactured and these patients were successfully treated.

Although these two trials are still in the patient follow-up stage, no serious adverse events have been observed suggesting that polyclonal Treg therapies are safe. We are now in the preparation stages of Phase II clinical trials in renal, liver, islet and heart transplant patients.

## The Relevance of Specificity for Treg Therapy

While the polyclonal expansion of Tregs using anti-CD3/antiCD28 beads is relatively straightforward and readily translatable into the clinic, extensive data from preclinical animal studies have demonstrated that the adoptive transfer of Tregs with direct or indirect allospecificity are superior to polyclonal Tregs at reducing graft rejection (56–58). However, the expansion of antigen-specific Tregs presents an additional set of parameters that need to be addressed, including the origin of APCs and the dose required. Initial studies by Taylor et al., used allogeneic splenocytes to enrich for murine allo-specific Tregs, which were more efficient at reducing graft versus host disease in a murine model compared to anti-CD3 stimulated Tregs (59). More recently, we have shown in a murine transplant model that Tregs need to have both direct and indirect allospecificities to induce indefinite survival of heart transplants (57). Furthermore, using a humanized transplant mouse model, in which immunodeficient mice were reconstituted with PBMCs, we show that human Tregs with direct allospecificity significantly reduced alloimmune-mediated injury of human skin grafts, when compared with polyclonal Tregs. As part of the ONE Study Consortium, Tregs with direct allospecificity are currently being evaluated at two different sites in the USA (NCT02244801 and NCT02091232). GMP regulations are far more rigorous in Europe compared to USA and the investigators have been able to use a standard cell sorter in their manufacturing process to purify Tregs, which is not possible in Europe. Few patients have been treated to date, but the completion of the trials will undoubtedly be very informative with regards to the safety of allospecific Tregs and provide possible clues on how well they compare against treatment with polyclonal Tregs.

In recent years, the transduction of chimeric antigen receptors (CAR) on T cells has shown great promise in the field of cancer cell therapy, particularly for B cell lymphomas where there is a clear target antigen such as the B lymphocyte antigens, CD20, and CD19. This has paved the way for its potential use in Tregs. We and others have shown that the expression of CAR in Tregs can potentially be used for the treatment of xeno-GVHD and allo-graft rejection (60–62). In a human skin xenograft transplant model, the adoptive transfer of CAR Tregs were more effective at alleviating the alloimmune-mediated skin injury caused by transferring allogeneic PBMCs compared to polyclonal Tregs. Recently, the US Food and Drug administration approved two CAR T cell therapies, the first one, Kymriah (Tisagenlecleucel) was developed for the treatment of patients up to 25 years of age with B-cell precursor acute lymphoblastic leukemia and Yescarta (axicabtagene ciloleucel) for the treatment of adults with refractory large B cell lymphomas. These approvals signify an important development and no doubt will pave the way for continued commercialization of cell therapies.

## Conclusion

A key breakthrough in the translational potential of Treg cell therapy was the demonstration that human Tregs could be successfully isolated and expanded *ex vivo* while maintaining immunoregulatory function. This has enabled the application of these cells in the clinic, leading to Treg adoptive transfer in phase I clinical trials of bone marrow transplantation and type I diabetes (63–66) and more recently in the setting of solid organ transplantation (67, 68). The success of these trials is reliant on a highly reproducible process for the sustained manufacture of autologous patient-derived Tregs. In the setting of solid organ transplantation, we are faced with the challenge of a more targeted approach to suppress the immune system, and as such efforts have focused on the expansion of allo-antigen specific Tregs for cell therapy application. Here, we have highlighted our experience to date. However, whether a generalized effect of immunosuppression by the adoptive transfer of polyclonal Tregs could potentially be diminished by more targeted Treg therapy requires further investigation. The major drawback of the two phase I clinical trials completed by us is that the isolation technique for regulatory T cells relies on first generation magnetic bead isolation. The inability of this technique to select cells based on stricter criteria (CD25^hi^) or multiple parameters (low expression of CD127) has led to the development of a GMP compliant FACS cell sorter (MACSquant Tyto cell sorter, Miltenyi Biotech). The validation of the MACSQuant Tyto and its GMP accreditation has now meant that we can isolate Tregs based on several markers, further enhancing the purity and quality of the infused product. The high level of purity achieved with the MACSQuant Tyto will allow us to generate donor-specific Tregs either by using donor-derived APC or by transduction with CAR. Finally, the selection of the most favorable Treg population, which will give the best therapeutic advantage, will likely be further enhanced by the advent of new technologies.

With scientific knowledge and technology rapidly advancing in the field, the future of Treg cell therapy is set to only progress further. As such, our ultimate aim of immune tolerance in transplantation is soon to become a reality.

## Author Contributions

GL, NS, and NG contributed to the conception, design, writing, and revision of the manuscript. ST, CS, LF, TV, and RL contributed to the writing, and revision of the manuscript.

## Conflict of Interest Statement

The authors declare that the research was conducted in the absence of any commercial or financial relationships that could be construed as a potential conflict of interest.
